# Theory of Mind in the Wild: Toward Tackling the Challenges of Everyday Mental State Reasoning

**DOI:** 10.1371/journal.pone.0072835

**Published:** 2013-09-12

**Authors:** Annie E. Wertz, Tamsin C. German

**Affiliations:** Department of Psychological & Brain Sciences, University of California Santa Barbara, Santa Barbara, California, United States of America; Boston College, United States of America

## Abstract

A complete understanding of the cognitive systems underwriting theory of mind (ToM) abilities requires articulating how mental state representations are generated and processed in everyday situations. Individuals rarely announce their intentions prior to acting, and actions are often consistent with multiple mental states. In order for ToM to operate effectively in such situations, mental state representations should be generated in response to certain actions, even when those actions occur in the presence of mental state content derived from other aspects of the situation. Results from three experiments with preschool children and adults demonstrate that mental state information is indeed generated based on an approach action cue in situations that contain competing mental state information. Further, the frequency with which participants produced or endorsed explanations that include mental states about an approached object decreased when the competing mental state information about a different object was made explicit. This set of experiments provides some of the first steps toward identifying the observable action cues that are used to generate mental state representations in everyday situations and offers insight into how both young children and adults processes multiple mental state representations.

## Introduction

One of the most complicated and interesting problems human beings face in their day-to-day lives is making sense of what others around them are doing. From the perspective of cognitive science, accomplishing this task is a truly remarkable achievement. Human behavior is unimaginably complex and essentially limitless. Yet, when we watch the people around us, we do not experience their behavior as a confusing string of disconnected actions. Instead, we effortlessly interpret their behavior in terms of a finite set of conceptual entities – mental states like desires and beliefs. This ability to interpret, predict, and explain the actions of others in terms of underlying mental states is theory of mind (ToM); a set of cognitive capacities that has been studied over the past three decades from many different perspectives, including comparative psychology [1], [2], cognitive development, [3]–[7], and cognitive neuroscience [8]–[12]. Much has been learned about ToM, but despite the extensive attention this topic has received, we have just begun to scratch the surface.

One task in particular has dominated research on mental state reasoning: the false belief task [3], [7]. In the standard version of the task, preschool children are told a story about Sally, who puts her ball in a basket and leaves the room. Then, a second character, Ann, moves Sally’s ball from the basket to the box without Sally’s knowledge. When Sally comes back into the room, children are told that Sally wants to get her ball and then asked where she will look for it. In order to correctly predict Sally’s action, children must use Sally’s incorrect (i.e., false) belief about the ball’s location. This task and others like it have become the gold standard for investigating ToM. But real life isn’t like the false belief task. People don’t generally go around announcing their intentions prior to acting, nor do they constrain their actions to neat circumstances that contain only a few objects in discrete locations.

This is not to say that the false belief task hasn’t been a valuable tool. The studies using this task (and its many variations) have lead to findings of immense importance. However, there is more to mental state reasoning than the false belief task [13] and a critical next step will be to move beyond thinking about ToM in terms of these tasks and begin mapping out how mental state reasoning is achieved in messy, complicated, and underspecified everyday situations; in other words, to begin considering the ecologically valid circumstances of mental state reasoning.

At a broad level, there are two routes to figuring out what someone else is thinking: what a person says (a verbal route) and what a person does (a behavioral route) – a point made throughout the ToM literature (e.g., [5], [14]–[18]). Here we will focus primarily on reasoning about mental states based on behavioral cues and the challenges posed by doing so. One fundamental problem with ecologically valid mental state reasoning is the huge variability in human behavior. People can do an infinite number of things, but not all actions will be relevant (e.g., an accidental action such as someone tripping on a crack in the sidewalk; [19]–[21]), and some will be interpreted differently given the constraints of the situation (e.g., [22], [23]). Nevertheless, a subset of behaviors will be reliable indicators of beliefs and desires [15]. These specific actions must be identified, along with the types of mental states that are attributed to individuals performing such actions.

A second problem is that a person’s actions at any one time can often be consistent with several different underlying mental states. If someone opens the refrigerator, they might want to get the milk, or the juice, or a beer, or simply to check the contents before a shopping trip. In fact, it is a perfectly ordinary occurrence for a person to want, for example, the milk and the juice simultaneously. Because everyday situations are fluid and events unfold rapidly, there is a lot of uncertainty inherent in mental state reasoning in these contexts. Therefore, the ability to attribute multiple possible mental states to an individual and assess the probability of each fitting with a given sequence of events is critical.

Taken together, these two problems each suggest a corresponding feature that is likely to be part of everyday ToM reasoning: (i) mental state representations should be generated in response to specific actions, and (ii) these representations should be generated even when the actions occur in the presence of mental state information derived from other sources. To be clear, we are not arguing that these are the only components involved in ToM operation in ecologically valid circumstances. To the contrary, everyday mental state reasoning is complex and involves many different components that will require extensive investigation. However, in order to get a handle on this problem, we are beginning by isolating and systematically testing one particular piece of the larger puzzle.

### Generating Mental State Content Based on Action

A large body of existing research has demonstrated that action information is indeed used to interpret agents’ behavior. Adults readily attribute mental states to other agents based on patterns of action (e.g., [24]–[28]), and preschool aged children explain simple search actions in terms of underlying mental states [29]–[34]. Even very young infants have sophisticated systems for interpreting action. They attribute goals to agents based on patterns of action [35]–[47] and attribute goals to failed or incomplete actions, ruling out alternate explanations that infants simply form expectations about test events based on general associative mechanisms [48]–[52]. Infants’ interpretation of action also extends to the attribution of beliefs. Non-verbal tasks, which necessarily rely on action patterns and situational cues, have demonstrated that infants across a variety of cultural contexts [53] attribute belief states to agents [4], [54]–[56].

The ability to interpret actions in terms of goals, desires, and beliefs, is complex and early emerging, and the existing research has contributed much to our understanding of the conditions under which mental states are attributed and the developmental time course of this ability. However, infant studies generally rely on methods that show the same action performed repeatedly and often in isolation (e.g., an actor reaching for the same object over and over; [47]), while the adult studies test complex action patterns that are the aggregation of many different simpler actions. In order to for ToM to operate effectively in everyday situations, repeated or isolated presentations of an action cannot be necessary to provoke the generation of mental states, nor should mentalistic interpretations be confined to complex action patterns. Rather, mental state representations should be generated when certain simple actions occur, even if those actions occur in the presence of mental state information derived from other sources (e.g., utterances, other actions, and situational factors).

There are undoubtedly many actions that will fall into this category, but here we focus on one specific action: a simple approach action. Previous experiments have demonstrated that approach is a powerful indicator of underlying mental states – such as intentions, desires, and beliefs – in many different contexts. This has been shown with the movement of specific body parts (e.g., a hand reaching for an object [26], [36], [42], [43], [45], [50]) and with an entire animate agent or person moving toward an object or a location (e.g., [30]–[34], [44], [56]). Given this, it seemed likely that an approach action may be used as a reliable cue to underlying mental state content, even in the presence of indicators of additional mental state content. In particular, we were interested in the effect of an approach action directed toward an object.

How an approach action may interact with the status of the object being approached is an interesting empirical question. It seems plausible that mental state inferences may differ for safe and dangerous objects, objects of drastically different size or value, etc. This question can (and should) be addressed by future studies. In the experiments presented here, the objects were all neutrally or positively valenced everyday objects that could be handled or used (e.g., teddy bear, toy fish [Experiments 1 and 2]; hairdryer, bagel [Experiment 3]).

### The Previous Investigation

We began to investigate this by looking at whether adult participants would attribute mental states to a character based on a simple approach action cue when the character already had a stated desire for a different object [57]. Adult participants read scenarios describing a character’s interactions with a target object. In these scenarios the character placed the target object in location 1, but it was subsequently moved to location 2 without the character’s knowledge. At the end of each story, the character approached location 1 searching for the target object (a similar structure to the false belief explanation tasks used with preschool children; [29]–[34]).

In the experimental condition – Approach Distracter Object (ADO) – there was a second distracter object in location 1. Therefore, when the story character searched in location 1 for the target object, the search action was incidentally directed at the distracter object, forming the approach action cue under investigation. The structure of these stories allowed (i) a clean presentation of the approach action cue, such that the only interaction the character had with the distracter object was to incidentally move towards it during her search for the target object, and (ii) the creation of a cover story about the character and the target object that provided the competing mental state information, which included a statement that the character’s intention was “to get her [target object].” The term “competing mental state information” will be used throughout to refer to mental state information derived from sources other than the approach action cue under investigation. In the current task structure, this is mental state information about the target object.

The control condition – Approach Empty Location (AE) – stories were identical to the experimental condition stories, except that the distracter object was in location 2. In this case, when the story character approached location 1 to search for the target object, she approached an empty location and her actions were only consistent with mental states about the target object. The AE control condition held all other aspects of the experimental condition stories constant (the presence of two objects, two agents, two locations, etc.) ensuring that any differences between the two conditions were attributable to the approach action cue.

To probe for the presence of mental state representations, participants were required to rapidly endorse or reject candidate explanations of the story character’s behavior. Each explanation referred to an underlying mental state (e.g., a desire for an object, a true or false belief about an object’s location). As predicted, across two experiments, adults consistently endorsed two types of mental state explanations about the distracter object in the ADO experimental condition: a desire for the distracter object and a true belief about its location. These explanations were consistently rejected in the AE control condition, ruling out the possibility that the result was being driven by aspects of the story other than the presence of the predicted approach action cue. Participants also endorsed mental state explanations about the target object – a desire for the target object and a false belief about its location – across both the ADO and AE conditions, indicating that they were tracking the competing mental state information as well.

Taken together, the results from the previous investigation indicated that adults generate mental state representations based on the approach action cue – a desire for the approached object and a true belief about its location – even when the story character had a stated desire for a different object.

### The Current Experiments

The goal of the current experiments was twofold. First, we aimed to take a developmental perspective and test how preschool aged children respond when the simple approach action cue is presented alongside competing mental state information. We chose this age because although recent studies suggest that infants possess ToM competencies (e.g., [4], [53]–[56]), there are still important developmental changes occurring across the 3 to 5 year age range that influence performance on mental state reasoning tasks (e.g., [6]). Therefore, it was unclear how preschoolers would handle more complex situations with multiple possible mental state interpretations. Accordingly, Experiment 1 tested preschool children using modified versions of the vignettes from the previous investigation [57] to examine whether young children would generate distracter object responses based on the approach action cue even in the presence of competing mental state information about the target object.

Second, we examined factors that influence the kinds of explanations produced in situations that are consistent with multiple mental state interpretations. This was done by manipulating whether or not the competing mental state information about the target object was explicitly stated. Previous work has demonstrated that verbal information can systematically affect responses in mental state reasoning tasks by highlighting particular aspects of the scenarios [58]–[60]. Therefore, we predicted that an explicit statement that the character was searching for the target object would highlight the mental state content about the target object and make it less likely that participants would include distracter object mental state content in their responses. This desire statement was always present in the previous investigation [57], so to investigate its effect we created two versions of the explanation tasks in the current experiments: one with the desire statement present and the other with the desire statement absent. We examined the effect of the desire statement on the explanation production task used with preschoolers (Experiment 1) and adults (Experiment 2), as well as the explanation endorsement task used in the previous investigation (Experiment 3).

There are a range of important debates concerning the proper characterization of mental state reasoning mechanisms including questions of their domain specificity (e.g., [61], [62]), their automaticity [63], [64], and whether there is continuity or discontinuity in their development (e.g., [6], [65]). Although these are important and interesting questions, the current research does not address any of them directly; we are concerned here only with whether there is evidence for the specific features proposed earlier that would enable mental state reasoning in ecologically valid situations. Providing such evidence will in turn serve to further constrain theories on each of these other important questions.

## Experiment 1

To examine whether preschool aged children (3- to 5-year-olds) would be sensitive to the approach action cue when competing mental state information is present, the explanation tasks from [57] were made more appropriate for use with young children by simplifying the language, adding illustrations, reading the stories aloud, and requiring children to provide their own explanation for the character’s search action. This mode of response was used because preschool children are susceptible to a “yes bias” [66], making the explanation endorsement task used with adults susceptible to false positives and therefore unsuitable. A similar free-response format was used successfully in previous studies of preschoolers’ ability to attribute underlying mental states based on a search action [29]–[34].

We manipulated whether the competing mental state information about the target object was made explicit by creating two separate versions of the ADO and AE stories. The desire-statement-present version of the stories included a verbal statement that the character came back into the room to get the target object (as in [57]; e.g., “She wants to play with her [target object]”). In the desire-statement-absent version, that statement was removed from the story text and the character’s desire for the target object was instead implied by the structure of the stories. Removing the explicit statement about the character’s desire for the target object provided the best circumstances for preschoolers to explain the character’s search by referencing mental states about the distracter object.

### Predictions

If preschoolers are sensitive to the approach action cue, they should produce mental state explanations referencing the approached distracter object, even though the stories are about the character’s interactions with the target object. Critically, distracter object explanations should be produced only in the ADO experimental condition (in which the approach action cue is present) but not in the AE control condition (which hold constant all aspects of the stories except the presence of the approach action cue). Therefore, we are predicting a significant condition difference such that preschoolers should produce significantly more distracter object responses in the ADO condition than the AE condition. If preschoolers are not sensitive to the approach action cue and only mention the distracter object because they confuse the two objects, or respond to the pragmatics of having their attention drawn to two objects in the stories, then they will produce mental state explanations referencing the distracter object in both conditions. If this is the case, there will be no difference in distracter object responses across the ADO and AE conditions.

Because the stories were designed to be about the character’s interactions with the target object and thereby provide the competing mental state information, preschoolers were also expected to produce explanations that referenced the target object. These were expected to be explanations referencing either a desire for the target object or a false belief about the target object – the same type of explanations that adults consistently endorsed in [57] and preschoolers produced in previous studies of mental state explanation [29]–[34]. Unlike distracter object explanations, preschoolers were expected to offer target object explanations equally across the ADO and AE conditions. Finally, preschoolers were expected to produce more mental state explanations referencing the distracter object in response to the desire-statement-absent stories than the desire-statement-present stories.

### Methods

#### Ethics Statement

This research was approved by the Human Subjects Committee at the University of California, Santa Barbara. Written informed consent was obtained from the parents of all participants.

### Participants

Eighty preschool children (33 females, 47 males; age in years: M = 4.46, SD = .62; range: 3.21–5.73 years) from the Santa Barbara, California area participated in this study. Three additional children were run, but excluded from the analyses because of failure to complete the task (N = 1) and experimenter error during the session (N = 2). Children were evenly divided between the desire-statement-absent and desire-statement-present versions of the stories.

### Design

**Figure 1:**
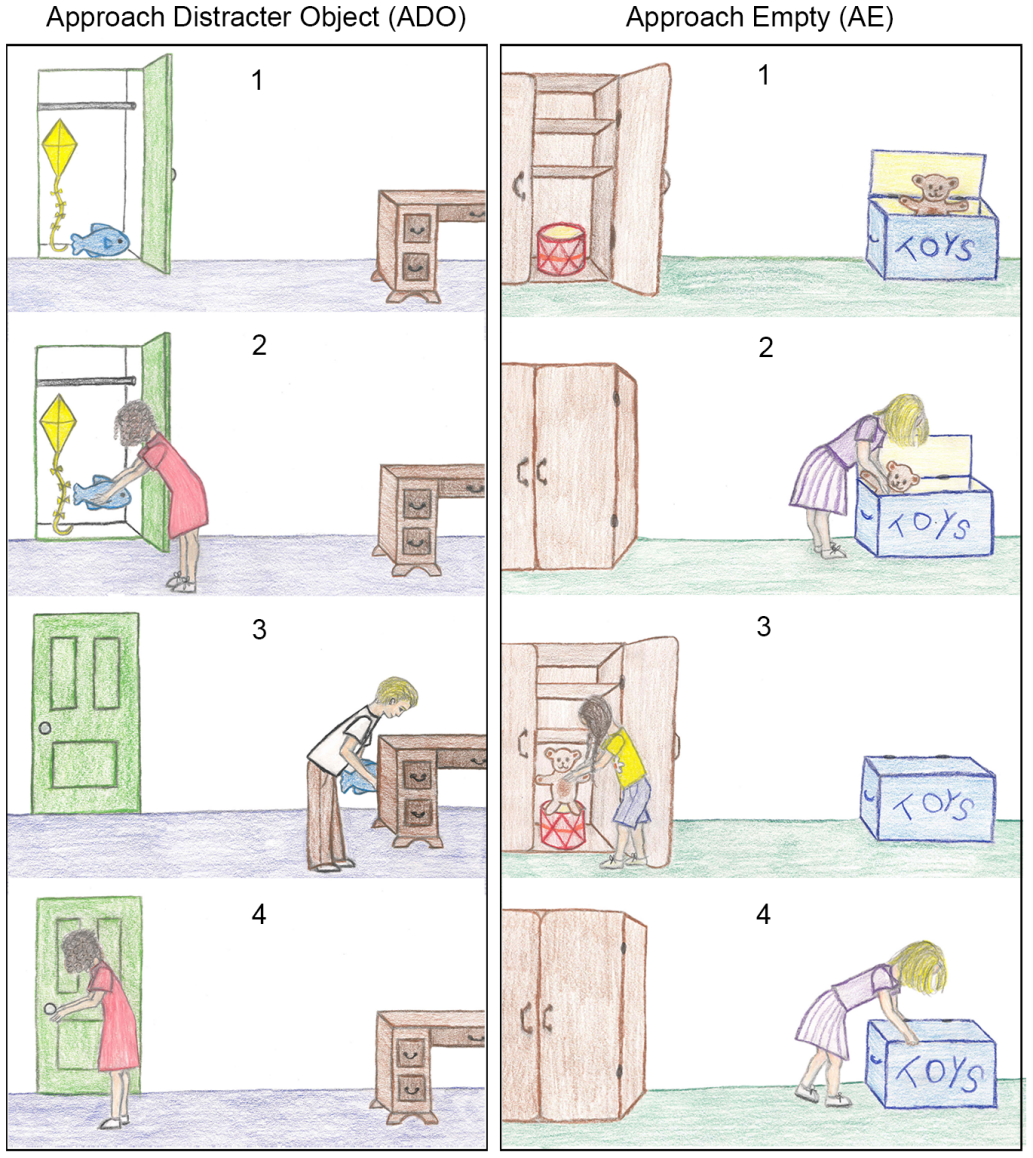
Task diagram for Experiments 1 and 2. Four of 10 total panels are depicted for each condition (see Figure S1 and Figure S2 for complete stories and Text S1 for the corresponding text). The ADO experimental condition is depicted on the left; the AE control condition is depicted on the right. The initial locations of both the target and distracter objects are shown (Panel 1). Next, character A returns the target object to its original location (Panel 2) after playing with it (a panel not shown here). Then, character B moves the target object to a different location (Panel 3). Finally, character A searches at the original location of the target object (Panel 4) and participants are asked “Why did she go there?” The initial location of the objects (left vs. right side) was counterbalanced across the four stories. Note that the only difference between the ADO and AE conditions is the location of the distracter object.

Each preschooler completed four explanation tasks: two ADO experimental stories and two AE control stories. Whether preschoolers heard the ADO or AE stories first was counterbalanced across participants. Each story included two agents, two objects, and two locations. In each story, character A placed a target object in location 1 and left the room. While character A was absent, character B moved the target object to location 2. Then, character A came back into the room and approached location 1 (see Figure 1). In the ADO experimental stories, a second distracter object was in location 1 so that when the character searched, she approached the distracter object, creating the simple approach action cue. In the AE control stories, everything was identical except that the distracter object was in location 2 so that the character approached an empty location during her search. See Figure S1, Figure S2, and Text S1 for a complete story from each condition.

The objects in both conditions were always occluded at the time of the character’s search. The side of the location at which the character searched (left vs. right side of the page) was counterbalanced across the four stories, as was the initial location of the objects. Half of the preschoolers saw desire-statement-present versions of the ADO and AE stories in which the statement “She wants to play with her [target object]” was included in the text of the stories. The other half saw desire-statement-absent versions of the ADO and AE stories in which that statement was removed from the text.

In order to compare their explanation task performance to more standard assessments of mental state reasoning, children were given a false belief action prediction task (Sally-Ann switched locations task; [3]) in a storybook format. This type of false belief task requires children to predict where a character will search for an object that has been moved without the character’s knowledge.

#### Procedure

Children were tested individually at a quiet table at their preschool by two experimenters. The first experimenter read the tasks aloud while showing children the events in a picture book. Children heard the four explanation stories first. At two points during each story, children were asked control questions to verify that they tracked the location of both the target and distracter objects (“Where is the [target/distracter object]?”). These questions were asked when the target object was placed in location 1, and after the target object was moved to location 2. If the child answered these questions incorrectly, the experimenter corrected the child by showing him/her the object’s location on a previous page, turning back, and asking the child the object location questions again. This was repeated until the child answered correctly to ensure that the child was keeping track of the objects’ locations.

The final page of each story contained only the last story panel, which showed the character approaching a location (panel 4 in Figure 1 above). While viewing this page, preschoolers were asked an explanation question (“Why did [character A] go there?”), a desire question (“What did [character A] want when she came back into the room?”), and a belief question (“Where did [character A] think the [target object] was?”). Each child was asked these questions in the same order. There were no explicit restrictions on the length or number of explanations that preschoolers could provide.

After completing the four explanation tasks, children were given the false belief action prediction task. Children were asked an action prediction question (“Where will she look for her ball?”), and two control questions (“Where was her ball in the beginning?” and “Where is her ball now?”). The second experimenter sat quietly throughout the testing sessions and wrote down the children’s responses to all questions.

### Results

#### What types of explanations do preschoolers produce?

The goal of this first analysis was to identify the explanations in which preschoolers attributed mental states to the character (i.e., the overall proportion of belief and desire explanations). In subsequent analyses, these mental state explanations were further divided by whether they referred to the target or distracter object. Children offered a few mental state explanations that did not reference a particular object (e.g., “She wanted to look inside”; 11.2% of desire explanations for the desire-statement-absent stories; 4.4% of desire explanations for the desire-statement-present stories); these types of explanations were offered equally across the ADO and AE conditions and did not factor into the subsequent analyses.

Preschoolers’ explanations were coded by two independent raters. Explanations were scored into three categories: Desire, Belief and Other. Desire explanations referred to the character’s motivation to search (e.g., “She wanted her bear,” “To get her bear”). Belief explanations referred to the character’s epistemic state, either explicitly mentioned (e.g., “She thinks her bear is in there,” “Cause he didn’t know it was over here”), or left implicit (i.e., making reference to the conditions giving rise to the epistemic state; e.g., “That is where she left the bear”). Previous studies of mental state explanations have used similar criteria for belief explanations (e.g., [33], [34]). All other explanations fell into the Other category (e.g., “There’s nothing in there,” “I don’t know”). Rater agreement was 96.9% for the desire-statement-absent version of the stories and 97.5% for the desire-statement-present version. The remaining coding discrepancies were resolved through discussion prior to analysis.

The frequency with which preschoolers produced each explanation type is listed in Table 1. There were no predictions about condition differences for these overall explanation types, and preliminary analyses confirmed that the proportion of these explanations did not differ across the ADO and AE conditions for either the desire-statement-absent or desire-statement-present versions of the stories, so the data are presented collapsed across condition. However, the presence of a verbally conveyed desire did systematically alter the types of mental state explanations that preschoolers produced. When the desire statement was absent, preschoolers produced more desire explanations than when the desire statement was present (z test for proportions, z = 6.51, p<.0001, φ = .36; the p-values reported throughout are two-tailed). The desire statement had the opposite effect on the number of belief explanations. Preschoolers produced many fewer belief explanations when the desire statement was absent than when it was present (z test for proportions, z = −5.10, p<.0001, φ = .29).

**Table 1:** Explanation types produced in Experiments 1 and 2.

Experiment	Story Version	Explanation Type		
	Desire Statement	Desire	Belief	Other
Exp. 1: Preschoolers (n = 80)	Absent (n = 40)	78.1%	11.9%	11.3%
	Present (n = 40)	42.5%	36.3%	21.3%
Exp. 2: Adults (n = 80)	Absent (n = 40)	61.9%	59.4%	1.3%
	Present (n = 40)	7.5%	94.4%	1.3%

Note: Percentages were calculated by dividing the number of explanations given in each category by the total number of explanations (160 total explanations). The percentages for each experiment total to greater than 100% because some of the explanations mentioned both a desire and belief.

#### Were preschoolers sensitive to the approach action cue, even in the presence of competing mental state information?

To assess whether preschool children attributed mental states to the character based on the approach action cue, we identified instances in which children cited mental states about the distracter object as the reason for the character’s search in each condition. If those mental states were attributed based on the approach action cue, such instances should occur in the ADO condition, but not the AE condition. Each child had two opportunities to reference mental states about the distracter object for each story: their explanation for the character’s search action and their answer to the desire question. Therefore, the analyses were conducted based on the number of children who produced distracter object mental state responses. Preliminary analyses indicated that there were no order effects, therefore the analyses presented below are collapsed across presentation order.

**Figure 2:**
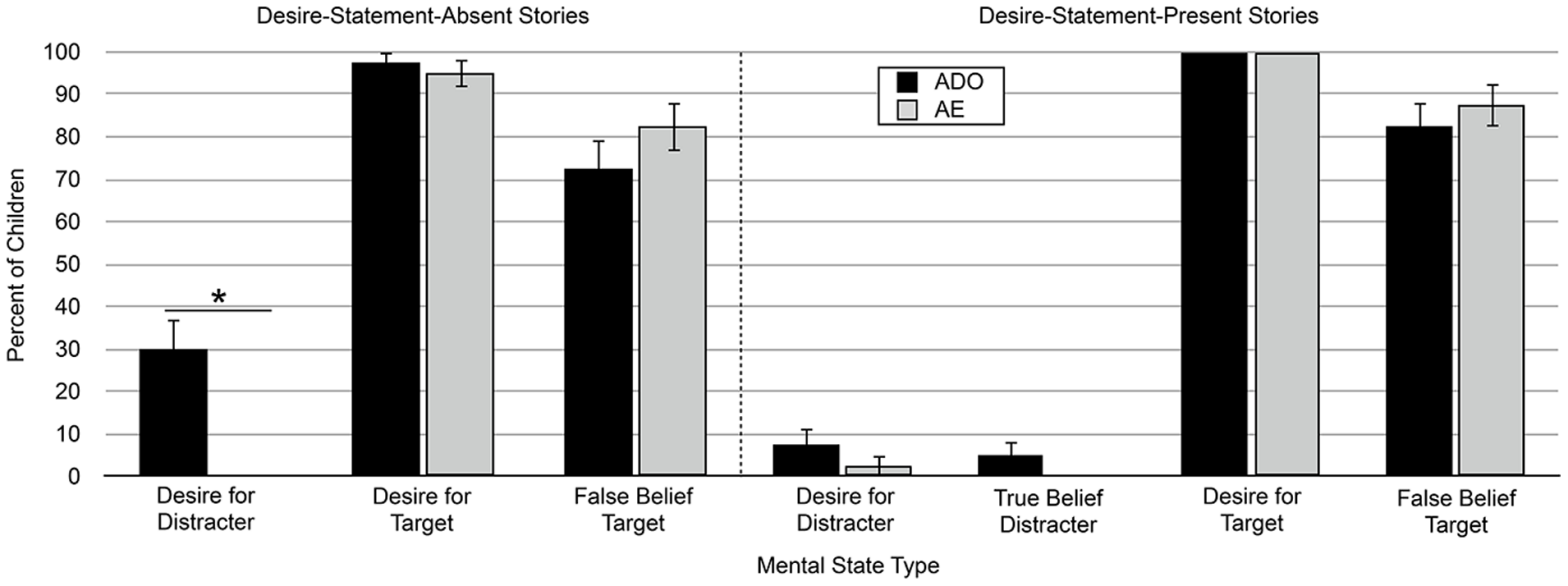
Percent of children producing each type of mental state response in Experiment 1. The “true belief distracter” responses are not shown for the desire-statement-absent stories because none were produced. Error bars represent the standard error of each percentage. * indicates McNemar test of dependent proportions, p<.0001, two-tailed.

As predicted, children who saw the desire-statement-absent version of the stories produced responses that included mental states about the distracter object in the ADO condition, but not the AE condition. In total, 30% of children attributed a desire for the distracter object to the character in the ADO condition, a significant difference from the 0% in the AE condition (McNemar test of dependent proportions, p<.0001; see Figure 2). Children who saw the desire-statement-present version of the stories also produced a limited number of distracter object responses. Although the number of children who referenced the character’s mental states about the distracter object was greater in the ADO condition than in the AE condition, the difference was not significant (ADO: 12.5% of children vs. AE: 2.5%, McNemar test, p = .22, see Figure 2 for distracter object mental states broken down by mental state type and condition: Desire for Distracter: ADO: 7.5% of children vs. AE: 2.5%, McNemar test, p = .625; True Belief about Distracter: ADO: 5% vs. AE: 0%, McNemar test, p = .50). Making explicit the competing mental state information about the target object resulted in a marginally significant decrease in the number of children who provided distracter object responses (χ^2^(1, N = 80)  = 3.66, p = .056).

There were no consistent age effects on the tendency to attribute mental states about the distracter object to the character. Children who mentioned the distracter object for the desire-statement-present version of the stories were younger (M = 4.19 years, SD = .49 years) than children who did not (M = 4.70 years, SD = .50 years; independent samples t-test, t(38)  = −2.27, p = .03, d = 1.00). However, for the desire-statement-absent version there was no significant age difference between children who mentioned the distracter object (M = 4.11 years, SD = .68 years) and those who did not (M = 4.39 years, SD = .65 years; t(38)  = −1.21, p = .23).

Taken together, these results suggests that (i) preschoolers are capable of using the approach action cue to generate mental state representations about the distracter object in the presence of competing mental state information, and (ii) explicitly stating the competing mental state information about the target object made it marginally less likely that distracter object mental state representations generated based on the approach action cue would enter into preschoolers’ explanations of the character’s search action.

#### Did preschoolers track the competing mental state information about the target object in both the desire-statement-present and desire-statement-absent versions of the stories?

To assess this, we identified instances in which children produced mental states about the target object. Preschoolers had two opportunities to produce a desire for the target object (the explanation question and the desire question) and two opportunities to produce a false belief about the target object (the explanation question and the belief question). As before, the analyses were based on the number of children who produced each type of target object response. Preliminary analyses again showed that there were no order effects. Unsurprisingly, 100% of children who saw the desire-statement-present version of the stories, in which the character’s desire for the target object was explicitly stated, attributed some type of target object mental state to the character in both the ADO and AE conditions (see Table 2). These mental states were a desire for the target object and a false belief about its location, and were attributed equally across the two conditions (see Figure 2 for target object mental states broken down by mental state type and condition: Desire for Target: ADO: 100% of children vs. AE: 100%, McNemar test, p = 1.00; False Belief about Target: ADO: 82.5% vs. AE: 87.5%, McNemar test, p = .50).

**Table 2:** Percent of participants who produced distracter object and target object responses in Experiments 1 and 2.

		Distracter Responses		Target Responses	
Experiment	Story Version	Condition		Condition	
	Desire Statement	ADO	AE	ADO	AE
Exp. 1: Preschoolers (n = 80)	Absent (n = 40)	30%	0%	97.5%	100%
	Present (n = 40)	12.5%	2.5%	100%	100%
Exp. 2: Adults (n = 80)	Absent (n = 40)	60%	0%	100%	100%
	Present (n = 40)	0%	0%	100%	100%

Note: The comparisons between preschool children and adults producing distracter object responses in the ADO condition were significant for the desire-statement-absent stories (Fisher’s Exact, P = .01) and marginally significant for the desire-statement-present stories (Fisher’s Exact, P = .055). None of the other age comparisons were significant.

Importantly, the same pattern held for the desire-statement-absent version of the stories, in which the character’s desire for the target object was strongly implied, but not explicitly stated. One hundred percent of children attributed some type of target object mental state to the character in the AE condition, and 97.5% of these same children attributed some type of target object mental state in the ADO condition (see Table 2). As before, these mental states were a desire for the target object and a false belief about its location. Children attributed these mental states equally across the two conditions (see Figure 2 for target object mental states broken down by mental state type and condition: Desire for Target: ADO: 97.5% vs. AE: 95%, McNemar test p = 1.00; False Belief about Target: ADO: 72.5% vs. AE: 82.5%, McNemar test, p = .13). Children clearly interpreted the desire-statement-absent stories as being primarily about the character’s interactions with the target object, even though these stories lacked an explicit statement of the character’s desire for the target object. Taken together, these results indicate that children were tracking the competing mental state information about the target object in both the desire-statement-present and desire-statement-absent versions of the stories.

#### Control question performance

Responses to the object location control questions (“Where is the [target/distracter object]?”) indicated that children correctly tracked the locations of the two objects throughout the stories. For the desire-statement-absent version of the stories, children were 95.3% correct on the location of the target object and 96.6% correct on the location of the distracter object. For the desire-statement-present version, children were 96.9% correct on the location of the target object and 94.4% correct on the location of the distracter object. Note that these numbers reflect children’s first responses to the location control questions; children were always required to answer correctly before continuing with the story.

Children were given an object location score based on the number of correct first responses to the object location question (scores could range from 0–8; based on two questions per story across four stories). This was compared to the total number of distracter object responses children produced (scores could range from 0–8; based on two opportunities per story across four stories). There was no relationship between providing distracter object mental state responses and children’s ability to correctly track the location of the distracter object for either version of the stories. However, there was a correlation between providing distracter object mental state responses and children’s ability to track the location of the target object. Children were more likely to mention the distracter object when their first answer to the location control question about the target object was incorrect (desire-statement-present stories: Pearson correlations, r(38)  = −.45, p = .004; desire-statement-absent stories: r(38)  = −.39, p = .01).

#### Comparisons with standard false belief task performance

Children’s performance on a standard false belief action prediction task was compared to whether or not they attributed a mental state about the distracter object to the character. Only children who passed both action prediction control questions were used in the analyses (desire-statement-absent stories: N = 32, 71.9% correct action prediction; desire-statement-present stories: N = 35, 62.9% correct action prediction). The relationship between standard false belief task performance and attributing a desire for the distracter object was different for the desire-statement-absent and desire-statement-present stories. In the desire-statement-absent version of the stories, children who passed the false belief task were less likely to attribute a desire for the distracter object (phi correlation, r_φ_ = −.38, p = .03). However, there was no relationship between passing the false belief task and attributing a desire for the distracter object to the character in the desire-statement-present version (phi correlation, r_φ_ = −.19, p = .29). The developmental changes responsible for standard false belief task performance do not appear to consistently predict children’s use of the approach action cue.

### Discussion

Children explained the character’s search actions by referencing both desire and belief information, as found by prior investigations of action explanation (e.g., [29]–[34]). One finding of note here was that the presence or absence of a statement of the character’s desire for the target object systematically altered the overall proportions of desire and belief explanations offered. When the verbal desire statement was present, children were more likely to explain the search action by citing the character’s belief. When the desire statement was absent, children were more likely to explain the search action by citing the character’s desire. This pattern, though not predicted explicitly under the principal hypotheses of interest here, makes sense in terms of what is known about conversational pragmatics (e.g., [67], [68]).

Briefly, providing new information to a speaker (rather than information established as already known by that speaker) is one means by which relevance in utterances can be maximized. So, when desire information has been made explicit in the final sections of the vignette (as in, “Mary’s come back into the room. She wants to play with her [target object]”) it is plausibly less relevant for an explanation to convey that desire than in a case where the desire has been left implicit (e.g., “Mary’s come back into the room”).

A further point here is that while belief and desire information combine to provide “reasons” for action (see e.g., [69]), desire information can be considered critical for motivation of action (e.g., that Mary will do anything at all) while epistemic information constrains the specific action that might follow (e.g., where Mary will go). Desire information might therefore be more critical to explain an action’s occurrence, and as a consequence ought to be provided in any situation where it has not already been made explicit [70]. While we know of no explicit investigations into whether children of this age are sensitive to considerations of relevance when selecting explanations in this kind of task, observing a similar shift in the pattern of adult explanations in response to the presence or absence of desire information would lead credence to this interpretation (see also [71] for evidence of relevance considerations in younger children using a non-verbal task). The results of Experiment 2 will bear on this issue.

Turning to the hypotheses of specific interest, the results of Experiment 1 demonstrate that under certain conditions, preschoolers, like adults in [57], attribute mental state information based on an approach action cue even in the presence of competing mental state information. Children attributed a desire for the distracter object to the character in the ADO test condition (in which the approach action cue was present), but not in the AE control condition (in which the cue was absent), of the desire-statement-absent stories. The AE condition held constant all other aspects of the stories and therefore rules out alternate explanations such as general confusion, the mere presence of two objects in the story, or the general task pragmatics of having an experimenter draw attention to two different objects. Therefore, features that enable mental state reasoning in more complicated situations that are consistent with multiple mental state interpretations appear to be present at least by the preschool years.

Yet there was a developmental difference between preschoolers in Experiment 1 and adults in [57]. Adults in [57] reliably endorsed distracter object mental states despite the presence of a statement of the character’s desire for the target object, while preschoolers in the current experiment did not reliably produce such responses in similar circumstances. Similarly, there were hints of developmental trends within our sample. Children who produced distracter object mental states tended to be (i) younger and (ii) less likely to pass the standard false belief task, although these results were limited to children who saw certain versions of the stories (the desire-statement-present for the former and the desire-statement-absent for the latter). These findings are intriguing and deserving of further study. Because the age difference was found only for the desire-statement-present stories (in which very few children produced distracter object responses and the predicted difference between the ADO and AE conditions was not statistically significant), and not for the desire-statement-absent stories, the current data do not allow us to make claims about whether age has a systematic effect on children’s use of the approach action cue in the 3- to 5-year-old age range. However, children’s developing cognitive resources (both conceptual and executive) must play an important role in their ability to handle representations of multiple mental states. Future investigations could address this question by examining a broader age range and including tasks that require tracking a character’s mental states about more than two objects, or the mental states of multiple characters (e.g., [72]).

There are also several possible reasons for the correlation between attributing a desire for the distracter object and performance on the standard false belief in the desire-statement-absent stories (i.e., children who failed the false belief task were more likely to cite the character’s mental states about the distracter object). Each possibility raises questions about the nature of the underlying cognitive system (or systems) that children are bringing to bear on this task. For example, it could be that children who fail the false belief task are defaulting to more basic action-based systems to interpret the character’s behavior (e.g., something like a “social perception system” that relies on biological motion cues, eye gaze, etc. to infer intentions [73]–[78]). Or, it could be that children use the same underlying conceptual system on both tasks, but some children may have less well developed inhibitory resources that impact their ability to solve the standard false belief task and make it more likely for them to answer with the most immediately available mental state content [17], [58]–[60], [79]–[81]. Under this interpretation, the distracter object mental states were more immediately accessible because the very last thing the character did was approach the location containing the distracter object. Either of these possibilities is also consistent with the finding that, all else equal, children tend to offer explanations for the character’s search action that reference desires instead of beliefs [29]–[34], [70]. Future studies will be necessary to examine these issues in detail.

Of immediate interest here is the difference between preschoolers in the desire-statement-present version of Experiment 1 and adults in [57]. Perhaps the difference is developmental; perhaps young children are failing to produce distracter object responses where adults would succeed. Or, perhaps the difference is a consequence of the type of explanation task used. Adult participants in [57] were required to rapidly endorse or reject explanations that were presented to them, while preschoolers in Experiment 1 were required to come up with their own explanations. Therefore, the difference could be because the explanation endorsement task is a more sensitive probe for multiple mental state representations than the explanation production task.

In order to test between these possibilities, adult participants in Experiment 2 were given the same illustrated scenarios used with preschool children in Experiment 1 and asked to freely provide explanations for the character’s search action. This experiment also served as an additional test of whether adults generate mental state representations based on an approach action cue in the presence of competing mental state information.

## Experiment 2

### Predictions

Adults were expected to produce explanations referencing the character’s mental states about the distracter object in the ADO experimental condition, but not the AE control condition (i.e., we predicted significantly more distracter object responses in the ADO than the AE condition). Because the stories were primarily about the character’s interactions with the target object, adults were also expected to produce explanations that reference mental states about the target object; target object explanations were expected to be offered equally across the ADO and AE conditions.

If the difference between preschoolers in Experiment 1 and adults in [57] was due to developmental differences, adults should produce mental state explanations that reference the distracter object for both the desire-statement-absent and desire-statement-present versions of the stories. However, if the difference was a consequence of the free response format, adults (like preschoolers) should produce fewer distracter object explanations in response to the desire-statement-present version than the desire-statement-absent version of the stories.

### Methods

#### Ethics Statement

This research was approved by the Human Subjects Committee at the University of California, Santa Barbara. Written informed consent was obtained from all participants.

#### Participants

Eighty undergraduates (47 females, 33 males; age in years: M = 18.81, SD = 1.30) from the University of California, Santa Barbara participated in the study for course credit. Participants were evenly divided across the desire-statement-present and desire-statement-absent versions of the stories.

#### Design

Adult participants read the same stories given to preschoolers in Experiment 1 (see Figures 1, S1, and S2). Each participant read all four stories; two ADO stories and two AE stories. The order of presentation (i.e., whether they read the ADO or AE stories first) was counterbalanced across participants. The pictures and text from the stories were arranged in a comic strip format and made into packets for participants to read. The story panels containing the object location control questions were not included (panels 5 and 8 in Figures S1 and S2). To prevent participants from viewing information on previous pages while providing their explanations, the final page of each story contained only the last story panel (panel 4 in Figure 1) and a half-page of space for the participant to write their explanation of the story character’s action.

Half of the participants read the desire-statement-present version of the stories, which included a statement of the character’s desire for the target object (“She wants to play with her [target object]”). The other half read the desire-statement-absent version of the stories, which were identical except that the statement of the character’s desire was removed.

#### Procedure

Adults were given a packet containing the four explanation stories with an instruction page on the front. The instruction page informed participants that the stories were designed to be appropriate for preschoolers and told them to read the stories carefully. Participants were also instructed not to turn back to previous pages at any point in the story. The explanation question (“Why did [character A] go there?”) was included on the last page of each story. Adults wrote their explanations for the character’s search action in a half-page space provided at the end of each story. As in Experiment 1, there were no explicit restrictions on the length or number of explanations participants could offer.

### Results

#### What types of explanations do adults produce?

In order to identify explanations with mental state content, adults’ explanations were coded by two independent raters into the same three categories used in Experiment 1: Desire, Belief, and Other. These mental state explanations were divided by whether they referred to the target or distracter object for subsequent analyses. Adults also offered a few mental state explanations that did not reference a particular object (e.g., “She wants something”; 5.1% of desire-statement-absent desire explanations; or “He expected that things were left unchanged”; 1.1% of desire-statement-absent belief explanations; 0.7% of desire-statement-present belief explanations); these types of explanations were offered equally across the ADO and AE conditions and did not factor into the subsequent analyses. Rater agreement was 97.5% for the desire-statement-present stories and 95.6% for the desire-statement-absent stories. The remaining coding discrepancies were resolved through discussion prior to analysis.

The frequency with which adults produced each type of explanation is shown in Table 1. As in Experiment 1, there were no differences in the proportion of these explanation types across the ADO and AE conditions. The stated desire information impacted the proportions of desire and belief explanations offered by adults in the same way it impacted the explanation types offered by preschoolers in Experiment 1. When the desire statement was removed, adults produced many more desire explanation (desire-statement-absent stories = 61.9% vs. desire-statement-present stories = 7.5%; z test for proportions, z = 10.22, p<.0001, φ = .57) and fewer belief explanations (desire-statement-absent stories  = 59.4% vs. desire-statement-present stories  = 94.4%; z test for proportions, z = −7.42, p<.0001, φ = .42).

#### Did adults produce mental state explanations based on the approach action cue in the presence of competing mental state information?

**Figure 3:**
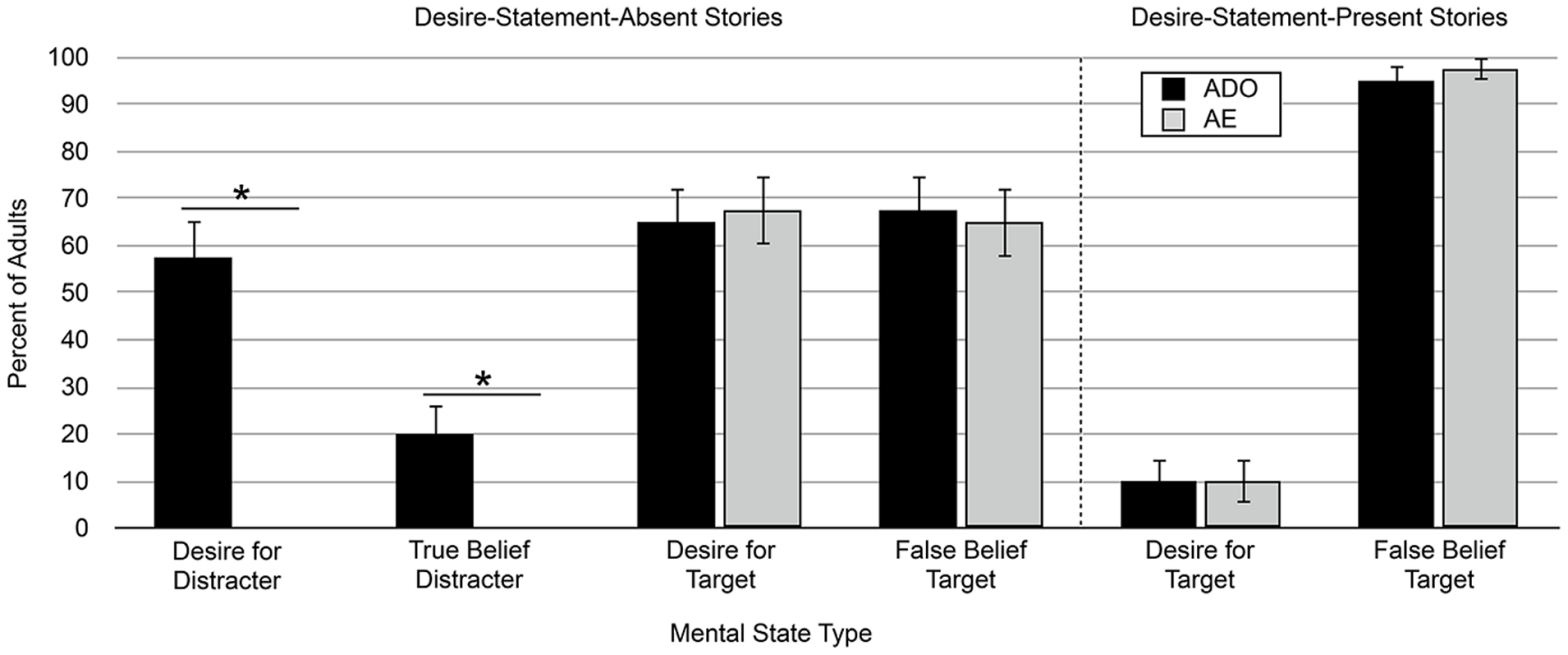
Percent of adults producing each type of mental state explanation in Experiment 2. Distracter object explanations are not shown for the desire-statement-present stories because none were produced. Error bars represent the standard error of each percentage. * indicates McNemar test of dependent proportions, p<.009, two-tailed.

As with the preschoolers, these analyses were based on the number of adults in each condition who cited a mental state about the distracter object as the reason for the character’s search. There were no order effects; the following analyses are collapsed across presentation order. Similar to the preschoolers, adults who read the desire-statement-absent stories readily produced explanations of the search action that referenced the character’s mental states about the distracter object. Although preschoolers only referenced the character’s desire for the distracter object, adults produced explanations referencing both a desire for the distracter object and a true belief about its location. Critically, adults only produced distracter object explanations in the ADO experimental condition, never in the AE control condition indicating that the approach action cue was used to generate mental states (see Figure 3 for distracter object mental states broken down by mental state type and condition: Desire for Distracter: ADO: 57.5% of adults vs. AE: 0%, McNemar test, p<.0001; True Belief about Distracter: ADO: 20% vs. AE: 0%, McNemar test, p = .008).

In total, 60% of adults attributed some type of mental state about the distracter object to the character in the ADO condition of the desire-statement-absent stories; significantly more than the number of preschoolers for these same stories in Experiment 1 (Fisher’s Exact, P = .01; see Table 2 for a comparison of preschoolers and adults). However, adults who read the desire-statement-present stories never produced distracter object explanations in either the ADO or AE conditions. This was marginally fewer adults than the number of preschoolers who produced distracter object responses for the same stories in Experiment 1 (Fisher’s Exact; P = .055); this same age comparison across the AE conditions of Experiments 1 and 2 was not significant (Fisher’s Exact; P = 1.00; see Table 2). The fact that adults did not produce distracter object explanations for the desire-statement-present stories suggests that the difference between preschoolers in Experiment 1 and adults in [57] is attributable (at least in part) to the free response format of the explanation production task. In this case, making the competing mental state information about the target object explicit reduced number of adults producing distracter object explanations to zero (desire-statement-absent stories: 60% vs. desire-statement-present stories: 0%; χ^2^(1, N = 80)  = 34.29, p<.0001).

#### Did adults track the competing mental state information about the target object in both the desire-statement-present and desire-statement-absent versions of the stories?

There were no order effects so the analyses are again collapsed across presentation order. For the desire-statement-present stories, which included a statement that the character was searching for her target object, 100% of adults offered some type of target object mental state explanation in both the ADO and AE conditions (see Table 2). As expected, adults offered these explanations equally across the two conditions (see Figure 3 for target object mental states broken down by mental state type and condition: Desire for Target: ADO: 10% of adults vs. AE: 10%, McNemar test, p = 1.00; False Belief about Target: ADO: 95% vs. AE: 97.5%, McNemar test, p = 1.00). Importantly, the same pattern held for the desire-statement-absent stories in which the statement of the character’s desire for the target object was removed; 100% of adults offered some type of target object mental state explanation in both conditions (see Table 2). Target object explanations were again offered equally across the ADO and AE conditions (see Figure 3 for target object mental states broken down by mental state type and condition: Desire for Target: ADO: 65% vs. AE: 67.5%, McNemar test, p = 1.00; False Belief about Target: ADO: 67.5% vs. AE: 65%, McNemar test, p = 1.00). Adults, like preschoolers in Experiment 1, clearly interpreted the stories as being primarily about the character’s interaction with the target object in both the desire-statement-present and desire-statement-absent stories, indicating that they were tracking the competing mental state information in both cases.

### Discussion

Adults provided explanations for the search action referencing the character’s desires and beliefs (see e.g., [30] for similar findings). Just as in Experiment 1, the presence or absence of explicitly stated desire information affected the relative proportions of belief and desire explanations offered, with more belief explanations being offered when the desire information was included immediately prior to the character’s search than when that desire information was omitted. As discussed above with respect to the preschool dataset (Experiment 1), this pattern make sense given considerations of relevance [67], [68], and the observation that adults showed the same pattern as did children lends weight to the speculation that relevance concerns may have driven this pattern in the younger participants.

Turning to the main hypotheses under test, adults who viewed the desire-statement-absent stories readily produced distracter object mental state explanations (a desire for the distracter object and a true belief about its location; the same explanation types endorsed by adults in [57]). As predicted, these explanations occurred in the ADO condition only, replicating the finding that a simple approach action is used to generate mental states in the presence of competing information. However, when the competing mental state information about the target object was made explicit (the desire-statement-present stories), adults failed to produce any distracter object mental state explanations. This is similar to the effect that this manipulation had on preschoolers in Experiment 1, suggesting that the difference between preschoolers in Experiment 1 and adults in [57] is, at least in part, a consequence of the free-response format used in Experiments 1 and 2 and not developmental differences alone. That is, when the competing mental state information about the target object was made explicit, adults in [57] reliably endorsed mental state information about the distracter object, but neither preschoolers (Experiment 1) nor adults (Experiment 2) reliably produced distracter object responses.

This is not to say that there were no developmental differences between preschoolers and adults in Experiments 1 and 2. The pattern of results was similar across the two age groups, but adults were more likely to produce distracter object responses for the desire-statement-absent stories than the preschoolers in Experiment 1. As we discussed for Experiment 1, the conceptual and processing resources that are brought to bear on these tasks must mature over the course of development and affect performance on these types of tasks. For example, adults may be better able to track the character’s mental states about multiple objects, or adults’ mature linguistic and working memory capabilities may enable them to produce more complex explanations that cite multiple reasons for the character’s search. In any case, perhaps unsurprisingly, adults seem more adept at reasoning about and explaining situations that are consistent with multiple mental state interpretations than are preschool children. Identifying the cognitive resources that are responsible for these changes will be an important task for future research.

Taken together, Experiments 1 and 2 indicate that preschoolers and adults generate mental state representations based on a simple approach action, even in the presence of competing mental state information; features that allow effective mental state reasoning in complex everyday situations. These results expand the findings of [57] while highlighting an interesting difference: adults in Experiment 2 did not produce distracter object explanations for the desire-statement-present versions of the stories, while adults in [57] reliably endorsed distracter object explanations under similar circumstances. This suggests that the explanation endorsement task may be a more sensitive probe for additional mental state content. Further, it is consistent with the idea that (at least for adults) distracter object mental states may be generated based on the approach action, but not necessarily included in participants’ responses under certain circumstances (a structure proposed by certain models of ToM reasoning; [5], [58], [82]–[86]).

To test this, Experiment 3 examined the effect of the desire statement on the more sensitive explanation endorsement task. Adult participants read stories that described the scenarios used in Experiments 1 and 2 in a text format. These stories were identical to those used in [57], except that the statement of the character’s desire for the target object was removed from the text, creating a desire-statement-absent version of the explanation endorsement task. The results were compared to the original results from the desire-statement-present version of the task used in [57].

## Experiment 3

### Predictions

If mental state representations are generated based on the approach action cue, but being blocked from inclusion in a response by the desire statement, participants in Experiment 3 should endorse distracter object mental state explanations (a desire and true belief) with greater frequency than participants in [57] did. This effect should be limited to the ADO condition; endorsement levels for distracter object explanations in the AE condition should remain the same.

### Methods

#### Ethics Statement

This research was approved by the Human Subjects Committee at the University of California, Santa Barbara. Written informed consent was obtained from all participants.

#### Participants

One hundred seventy nine undergraduates (129 females, 50 males; age in years: M = 18.95, SD = 1.39) from the University of California, Santa Barbara participated in this study for course credit.

### Design

Participants were divided across two separate experiments: Experiment 3A tested for the attribution of a desire for the distracter object (N = 90) and Experiment 3B tested for the attribution of a true belief about the distracter object’s location (N = 89). Within each of these experiments, participants were randomly assigned to either the ADO experimental condition (Experiment 3A: N = 43, Experiment 3B: N = 47) or the AE control condition (Experiment 3A: N = 47; Experiment 3B: N = 42). All participants read a series of 40 stories that described, in a text format, the tasks presented in Experiments 1 and 2 (see Table 3).

**Table 3:** Story structure used in Experiment 3.

Condition	
ADO	AE
Mary puts her [target object] in [location A] next to her [distracter object] and leaves the room. While Mary is away, Gina moves the [target object] to [location B]. Mary comes back into the room. She goes directly to [location A].	Mary puts her [target object] in [location A] and leaves the room. While Mary is away, Gina moves the [target object] to [location B] next to her [distracter object]. Mary comes back into the room. She goes directly to [location A].

Note: The difference between the ADO and AE conditions is italicized.

The text stories were identical to the stimuli used in [57] except that the sentence portion stating the character’s desire for the target object was removed (e.g., the italicized portion of the following sentence: “Mary comes back into the room to get her [target object]”). After reading each story, participants were presented with a candidate explanation for the character’s search action and required to rapidly endorse or reject it as a correct explanation of the character’s search. Four types of candidate explanations were assessed in each experiment; each referring to a mental state that could be attributed to the story character. In Experiment 3A (desire-for-distracter probe) two candidate explanations referenced possible desires: a desire for the distracter object and a desire for the target object. The other two explanations referenced possible beliefs about the target object: a false belief about the location of the target object (a belief the character held) and a true belief about the location of the target object (a belief the character never held). The candidate explanation types are shown in Table 4.

**Table 4:** Candidate explanation types used in Experiment 3A, desire probe.

Explanation Type	Example
Desire for Distracter	She wanted to get her [distracter object] from [location 1].*
Desire for Target	She wanted to get her [target object] from [location 1].
False Belief – Target	She thought her [target object] was in [location 1].
True Belief – Target	She thought her [target object] was in [location 2].

* Denotes the explanation type predicted to be endorsed in the ADO condition, but not in the AE condition.

In Experiment 3B (true-belief-about-distracter probe) all of the explanations were framed as beliefs (Table 5). Two of the explanations referenced beliefs that the character could possibly hold: a true belief about the distracter object and a false belief about the target object. The remaining two explanations referenced beliefs that the story character could not hold given the structure of the stories: a true belief about the target object and an impossible belief about the distracter object. The designation “impossible belief” refers to a belief that neither the participant nor the story character could hold based on the story information. This is an important distinction in that it preserves the designations “true” and “false” for beliefs that could plausibly be held by either the story character or the participant. Consider the true belief about the target object. Based on the information presented in the story, this is a belief that the participant can hold, but the story character cannot. In contrast, neither the participant nor the story character could hold the impossible belief about the target object based on the information in the story. Participants saw only one explanation per story, such that each explanation type was assessed ten times.

**Table 5:** Candidate explanation types used in Experiment 3B, belief probe.

Explanation Type	Example
True Belief – Distracter	She thought her [distracter object] was in [location 1].*
False Belief – Target	She thought her [target object] was in [location 1].
True Belief – Target	She thought her [target object] was in [location 2].
Impossible Belief – Distracter	She thought her [distracter object] was in [location 2].

* Denotes the explanation type predicted to be endorsed in the ADO condition, but not in the AE condition.

Note: The structure shown for the True Belief – Distracter and Impossible Belief – Distracter explanations applies to the ADO condition. The locations were changed to [location 2] and [location 1] respectively for the AE condition.

#### Procedure

The 40 tasks were presented in random order via E-Prime (Psychology Software Tools, Inc.) software. Each task began with the presentation of the story on the computer screen. Participants read the story at their own pace before pressing a key to advance. Next, an explanation question was displayed for two seconds (e.g., “Why does Mary go there?”), followed immediately by a screen displaying one candidate explanation. Participants pressed a green key to endorse the explanation as correct or a red key to reject it as incorrect. Participants were instructed to make these judgments as quickly as possible. Responses and response latencies were collected. Two practice trials preceded the 40 test trials.

### Results

The data analysis for this experiment was twofold. First, the data from Experiments 3A and 3B were analyzed to examine whether, in this task, adults use the approach action cue to generate mental states about the distracter object in the presence of competing mental state information about the target object. Next, mean endorsement levels for the distracter object explanations were compared to the mean endorsement levels from [57]. This second set of analyses addressed the focused question of whether removing the statement of the character’s desire for the target object would impact the frequency of distracter object mental state responses in the endorsement task as it did in the production task.

#### Experiment 3: Did adults attribute a desire for and a true belief about the distracter object to the story character based on the approach action cue?

These analyses were based on participants’ endorse/reject responses for each of the four explanation types in Experiments 3A and 3B. Each participant was given a score that reflected how many times (out of ten), they endorsed a particular explanation type as a correct explanation of the character’s search action. These scores were analyzed using a repeated measures ANOVA on condition (ADO vs. AE) and explanation type (Exp. 3A: desire for distracter, desire for target, false belief-target, true belief-target; Exp. 3B: true belief-distracter, false belief-target, true belief-target, and impossible belief-distracter). In both Experiments 3A and 3B, there was a main effect of condition (Exp. 3A: F(1, 88)  = 17.97, p<.0001, η^2^_p_ = .17; Exp. 3B: F(1, 87)  = 26.74, p<.0001, η^2^_p_ = .24; we report partial η^2^ throughout) and a main effect of explanation type (Exp. 3A: F(3, 264)  = 488.39, p<.0001, η^2^_p_ = .85; Exp. 3B: F(3, 261)  = 428.95, p<.0001, η^2^_p_ = .83), qualified by a condition by explanation type interaction (Exp. 3A: F(3, 264)  = 24.48, p<.0001, η^2^_p_ = .22; Exp. 3B: F(3, 261)  = 73.07, p<.0001, η^2^_p_ = .46).

**Figure 4:**
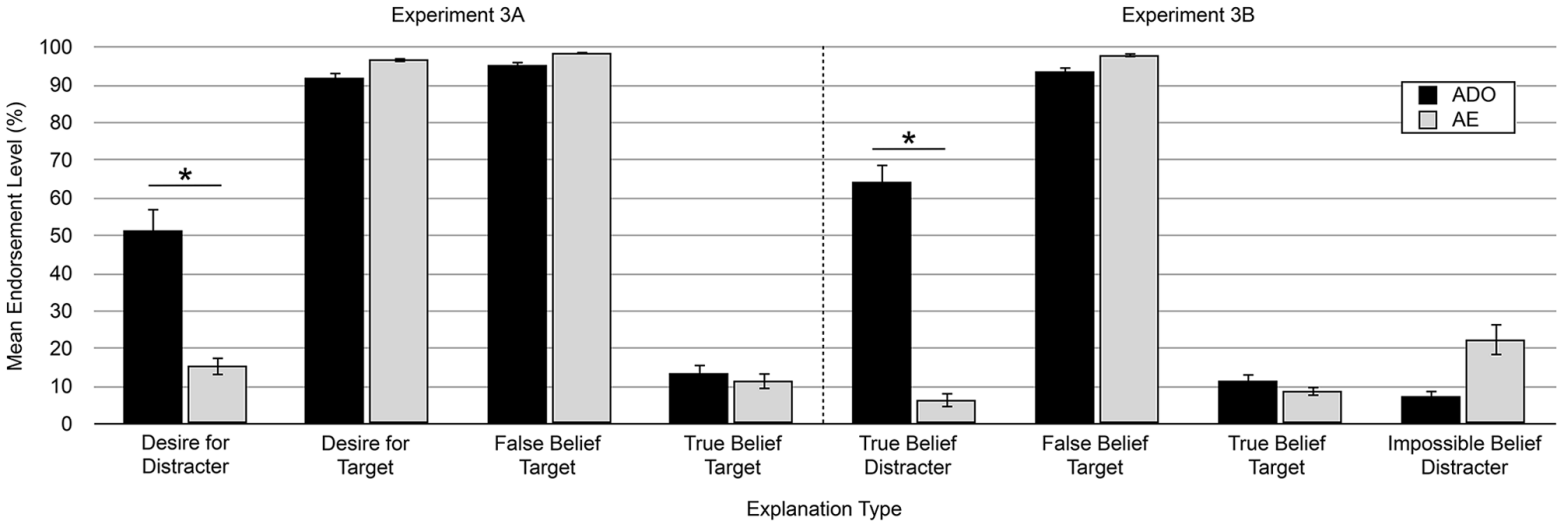
Mean endorsement level for the explanation types in Experiment 3. Error bars represent ±1 standard error of the mean (S.E.M). * indicates that the predicted difference was significant at p<.0001, two tailed; additional significant differences are reported in the main text.

As predicted, participants in Experiment 3A endorsed the desire for distracter explanations at significantly higher levels in the ADO condition (M = 51.4%) than in the AE condition (15.5%; independent samples t-test. t(88)  = 5.69, p<.0001, d = 1.20), and participants in Experiment 3B endorsed the true belief about distracter object explanations at much higher levels in the ADO condition (M = 64.3%) than the AE condition (M = 6.4%; t(87)  = 10.57, p<.0001, d = 2.24; see Figure 4). There were also differences across conditions in four other explanation types: two in Exp. 3A (desire for target: t(88)  = −2.65, p = .009, d = .56; false belief-target: t(88)  = −2.32, p = .02, d = .49), and two in Experiment 3B (false belief-target: t(87)  = −2.45, p = .02, d = .52; impossible belief-distracter: t(87)  = −3.43, p = .001, d = .73). However, the differences were in the opposite direction and smaller in magnitude than the predicted difference for the distracter object explanations. Taken together, Experiments 3A and 3B show that adults used the approach action cue to attribute a desire for the distracter object and a true belief about its location to the story character in the presence of competing mental state information, replicating the findings of [57].

The response time data from Experiments 3A and 3B were also analyzed with a repeated measures ANOVA on explanation type. Outliers (defined as ±3 standard deviations from the mean) were removed prior to analysis. Preliminary analyses showed no effect of condition, therefore the data were collapsed across condition for the reaction time analyses. In both experiments, there was a main effect of explanation type (Exp. 3A: F(3, 267)  = 29.67, p<.0001, η^2^_p_ = .25; Exp. 3B: F(3, 264)  = 13.90, p<.0001, η^2^_p_ = .14) such that endorsement of “correct” explanations was faster than rejection of “incorrect” explanations (Exp. 3A: desire for distracter: M = 2184.06 ms, SD = 603.31, desire for target: M = 1948.61 ms, SD = 485.62, false belief-target: M = 1773.41 ms, SD = 492.42, true belief-target: M = 2030.05 ms, SD = 506.77; Exp. 3B: true belief-distracter: M = 1998.58 ms, SD = 608.96, false belief-target: M = 1722.44 ms, SD = 431.44, true belief-target: M = 1949.63 ms, SD = 557.26, impossible belief-distracter: M = 1943.35 ms, SD = 566.28). In this case, “correct” explanations were those that were consistent with the competing mental state information about the target object. This was the same pattern found in [57].

#### Did the statement of the character’s desire for the target object affect the explanation endorsement task?

The main question addressed by Experiment 3 was whether the statement of the character’s desire for the target object affected adults’ responses in the explanation endorsement task as it did in the explanation production task: by blocking mental states about the distracter object from being included in a response. If so, participants in Experiment 3 (desire-statement-absent) should endorse explanations referencing mental states about the distracter object with greater frequency than did participants in [57] (desire-statement-present). A direct comparison of the distracter object explanations across the two experiments confirmed that, as predicted, participants in Experiment 3 endorsed distracter object explanations at significantly higher levels than participants in [57] (note that [57] is labeled as “Wertz & German” in the statistical analyses below). We report the results of [57] here as “mean endorsement level.” However, in the original paper, the results were reported as “errors,” where an error was defined as any response that was inconsistent with the overtly stated goal of the story character (see Figure 1 of [57]). “Mean endorsement level” for the data from [57] was calculated as described above and was chosen to be consistent with the reporting of the explanation production data.

Mean endorsement levels were higher for both distracter object explanation types in the ADO condition: desire for distracter object (Exp. 3A: M = 51.4%; Wertz & German, Exp. 1: M = 32.1%; t(75)  = 2.25, p = .03, d = .52) and true belief about distracter object (Exp. 3B: M = 64.3%; Wertz & German, Exp. 2: M = 42.4%; t(83)  = 2.83, p = .006, d = .62). Critically, neither of these same comparisons reached significance in the AE condition: desire for distracter object (Exp.3A: M = 15.5%; Wertz & German, Exp.1: M  = 13.9%; t(76) = .37, p = .71) and true belief about distracter object (Exp.3B: M = 6.4%; Wertz & German, Exp. 2: M  = 2.9%; t(78) = 1.46, p = .15. As predicted, the effect of removing the desire statement was limited to the ADO condition.

#### Cross-study comparisons of the additional explanation types

These comparisons revealed that the impact of removing the statement of the character’s desire for the target object was largely limited to the predicted distracter object explanations. Out of twelve additional comparisons across Experiment 3 and Wertz and German (see Figure 1 of [57]), there were only three significant differences. The first was found between Experiment 3A and Wertz & German, Experiment 1: endorsement levels for the false belief-target explanations were slightly elevated in the AE condition of the desire-statement-absent stories (Exp. 3A: M = 98.5%; Wertz & German, Exp. 1: M = 95.2%; t(76)  = 2.40, p = .02, d = .52). The two remaining differences were found between Experiment 3B and Wertz and German, Experiment 2. Endorsement levels for the true belief-target explanations were elevated in the ADO condition of the desire-statement-absent stories (Exp. 3B: M = 11.5%; Wertz & German, Exp. 2: M = 4.7%; t(83)  = 2.56, p = .01, d = .55) and endorsement levels of the impossible belief-distracter explanations were elevated in the AE condition of the desire-statement-absent stories (Exp. 3B: M = 22.4%; Wertz & German, Exp. 2: M = 10.3%; t(78)  = 2.29, p = .03, d = .51).

### Discussion

The explicit statement of the character’s desire for the target object had the same effect on the explanation endorsement task as it did on the explanation production task: decreasing the frequency of distracter object responses. In the current experiment, when the statement of the character’s desire for the target object was removed, adults endorsed mental state explanations about the distracter object with significantly greater frequency. This suggests that distracter object mental state representations are generated in the cognitive system based on the approach action cue, but are not always selected to be part of a given response (see e.g., [5], [58], [82]–[86]).

## General Discussion

Reasoning about others’ mental states in everyday situations poses two problems that must be overcome: (i) individuals do not often announce their intentions or beliefs, so mental states must instead be inferred based on what an individual does, and (ii) an individual’s actions can often be consistent with multiple underlying mental states. Therefore, in order for ToM to be effective in such situations, mental state representations should be generated in response to specific actions, even when those actions occur in the presence of mental state content derived from other sources.

The experiments presented here provide evidence for these design features by extending the findings of [57] to investigate (i) the developmental time course of the use of an approach action cue along side indicators of additional mental state information, and (ii) the factors that influence the kinds of explanations produced in situations that are consistent with multiple mental state interpretations. Experiment 1 provided clear evidence that preschoolers are capable of using a simple approach action to generate mental state representations in the presence of competing mental state information. This result is consistent with the burgeoning infancy literature showing that the ability to interpret actions in terms of underlying mental states is complex and early emerging (e.g., [4], [35]–[56]) and demonstrates that by the time children are four years old, isolated and/or repeated presentations of an action – standard features of infant studies – are not necessary. Rather, a single presentation of an approach action, even when it is presented as an incidental part of a larger story, is sufficient to prompt the generation of mental state representations.

Experiment 1 also uncovered an intriguing difference between preschoolers and adults in [57]: adults in [57] endorsed explanations citing mental states about the distracter object despite the character’s stated desire for the target object (desire-statement-present stories), while preschoolers only produced such responses at statistically significant levels when this statement was removed (desire-statement-absent stories). However, Experiment 2 – by exploring adults’ production responses and showing that these were similar to preschoolers’ – suggested that this difference could be traced (at least in part) to the different task demands of the explanation production and endorsement tasks, and not to more general developmental factors alone. In Experiment 2, adults, like preschoolers, reliably produced distracter object explanations only when the statement of the character’s desire for the target object was omitted from the text (desire-statement-absent stories).

While adults never produced distracter object explanations when the competing mental state information about the target object was made explicit, they reliably endorsed such explanations under similar circumstances in [57], indicating that the endorsement task is a more sensitive probe for multiple mental state representations. Nevertheless, Experiment 3 showed that the desire statement affected the endorsement task the same way it affected the production task – by selectively decreasing distracter object responses. When the desire statement was removed in Experiment 3, adults endorsed mental state explanations about the distracter object at significantly higher levels than in [57] when the desire statement was present. This suggests that mental states based on the approach action cue may be generated, but not always included in responses, a mode of operation proposed by certain models of mental state reasoning [5], [58], .

### Being Precise about Ambiguity

An apparent alternate interpretation of our findings is that participants only produced distracter object responses because the experimental scenarios were ambiguous or created confusion, not because of the approach action cue per se. While it may be true that participants subjectively experienced more uncertainty in the experimental scenarios (i.e., the reason for the search in the ADO condition may have seemed less clear cut), the question of why participants would experience confusion only in the ADO experimental condition and not the AE control condition must be addressed. In order to account for this selectivity, one must grant that ambiguity is only created when the distracter object is approached – this is, after all, the only difference between the ADO and AE conditions. Therefore, we argue that rather than being an alternative explanation, ambiguity is instead the phenomenological consequence of the additional mental state representations generated in response to the approach action cue in our scenarios.

### Implications for Theory of Mind

Taken together, the present experiments, along with the findings of [57], provide evidence for features that enable ToM to operate effectively in complex and unconstrained everyday situations: preschoolers and adults generate mental state representations based on a simple approach action that occurs in the context of competing mental state information. Yet these findings are only the initial steps toward untangling the cognitive processes that enable ecologically valid mental state reasoning; there is much that remains to be discovered. For example, while the approach action cue is clearly important, it is certainly not the only cue used to generate mental state representations. An important task for future investigations will be to identify additional action cues, the range of communicative cues, both verbal (e.g., stating one’s belief or desire) and non-verbal (e.g., pointing to or signaling via a sign, [63], [64]), and the corresponding mental state representations that are generated when such cues are present – what we might call adopting a “cue based approach” (see e.g., [87], ) to ToM reasoning.

Additionally, in the current studies, we investigated the approach action cue in contexts in which it is directed toward a location containing an object. It is an interesting theoretical question whether the use of the approach action cue would be different when it is directed toward a location where a character (incorrectly) thinks there is an object. In fact, it is possible that this version of the cue was driving mental state explanations about the target object in the desire-statement-absent stories. That is, the target object responses for those stories might have been offered because the character approached a location where she (falsely) believes the target object is located. However, the current set of experiments do not allow us to determine what factors are driving target object mental state explanations because the character has many different interactions with the target object throughout the scenarios. Therefore future studies will be necessary to tease apart the effect of an approach action directed toward a location that contains an object compared to a location where a character only thinks an object resides.

The current results are consistent with existing models of mental state reasoning that conceptualize the ToM system as generating multiple mental state representations for a given situation, of which only a subset are selected to be included in a response (e.g., [5], [58], [82]–[86]). Similarly, the robust effect of manipulating the presence or absence of a statement of the character’s desire for the target object across two different explanation tasks adds to previous demonstrations that verbal information can systematically affect responses in mental state reasoning tasks (e.g., the “look first” manipulation; [58]–[60]). However, future studies will be needed to explore whether the mental state reasoning processes uncovered here are the output of domain specific or more domain general mechanisms. Given the complexity of ecologically valid mental state reasoning, we suspect that many different mechanisms will be brought to bear on the problem, some of which may be specialized for mental state reasoning while others may be engaged by a broader array of tasks. The challenge going forward will be to develop models of mental state reasoning that can account for this complexity.

Additional investigations will also be necessary to uncover other features of the underlying cognitive processes identified in the current studies. For example, the current results address an outstanding question from [57] of whether participants generate distracter object explanations on their own, or only endorse them after the fact. Clearly participants freely produce the same kinds of mental state explanations that were endorsed in [57] based on the approach action. However, the timing of distracter object mental state generation is still unclear (i.e., whether such representations are generated “online” or “offline;” e.g., [89], [90]). The mental state representations based on the approach action could be generated either (i) at the moment the approach action occurs, or (ii) retroactively in response to being asked to explain the character’s behavior (in either free response or endorsement tasks). The outcome of studies investigating these (and other) questions will help to further elucidate the nature of the underlying mechanisms engaged by ecologically valid mental state reasoning.

## Conclusions

Determining how mental state reasoning is carried out in ecologically valid circumstances – messy, rapidly changing, underspecified contexts in which an individual’s actions may be consistent with multiple underlying mental states – is a fascinating problem. The experiments presented here provide a few tentative steps toward identifying the observable action cues that are used to generate mental state representations in everyday situations, and offer insight into how both young children and adults processes multiple mental state representations. This is a difficult task, as even the simple scenarios used in the current experiments isolating only one facet of everyday mental state reasoning contain multiple cues – both action based and verbally conveyed – and highlight the complexity of the computational processing required to handle just one small sliver of this kind of mental state reasoning. Nevertheless, demonstrating that preschool children and adults use an approach action cue to generate mental states in the presence of competing mental state information is an important step toward mapping the design of theory of mind.

## Supporting Information

Figure S1 **Complete Approach Distracter Object (ADO) experimental story.** All ten panels and accompanying text are pictured. Note that this the text used for the desire-statement-present version of the stories; the text for the desire-statement-absent version of the stories was identical, except that the phrase “She wants to play with her [target object]” on panel 9 was removed. See Text S1 for the complete text from both versions of this story.
(TIF)

Figure S2 **Complete Approach Empty Location (AE) control story.** All ten panels and accompanying text are pictured. Note that this the text used for the desire-statement-present version of the stories; the text for the desire-statement-absent version of the stories was identical, except that the phrase “She wants to play with her [target object]” on panel 9 was removed. See Text S1 for the complete text from both versions of this story.
(TIF)

Text S1 **Full text of the explanation task stories depicted in Figures S1 and S2.** The numbers refer to the story panels on which the text appears. The italicized text on panel 9 was included in the desire-statement-present version of the stories and removed from the desire-statement-absent version.
(DOC)
