# Supplementary figures and images for: Theory of Mind in the Wild: Toward Tackling the Challenges of Everyday Mental State Reasoning
Source: PLoS One. 2013 Sep 12;8(9):e72835. doi: 10.1371/journal.pone.0072835 (PMC3771964; doi:10.1371/journal.pone.0072835)

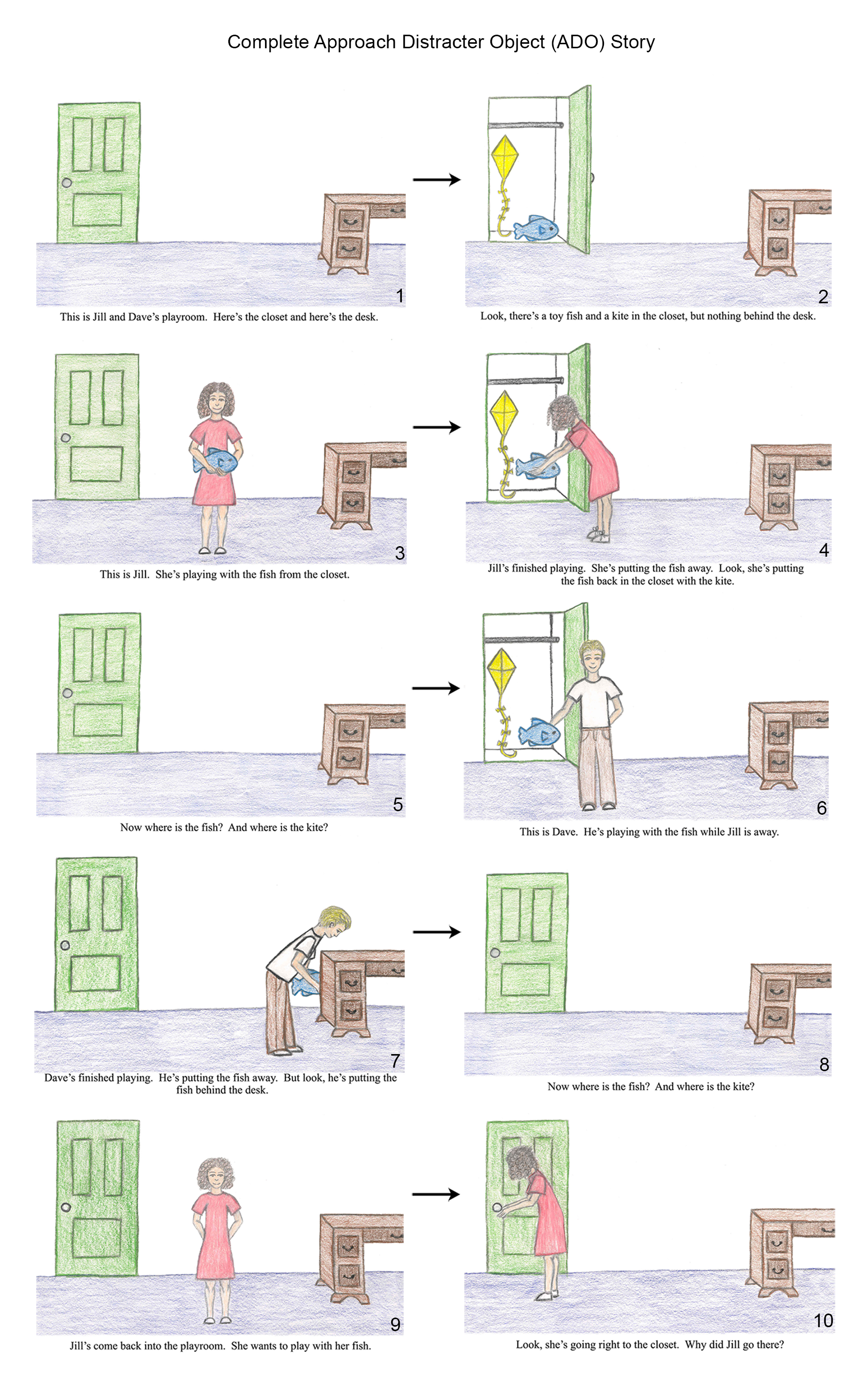

Supplement: Figure S1 — Complete Approach Distracter Object (ADO) experimental story. All ten panels and accompanying text are pictured. Note that this the text used for the desire-statement-present version of the stories; the text for the desire-statement-absent version of the stories was identical, except that the phrase “She wants to play with her [target object]” on panel 9 was removed. See Text S1 for the complete text from both versions of this story. (TIF) [file pone.0072835.s001.tif]

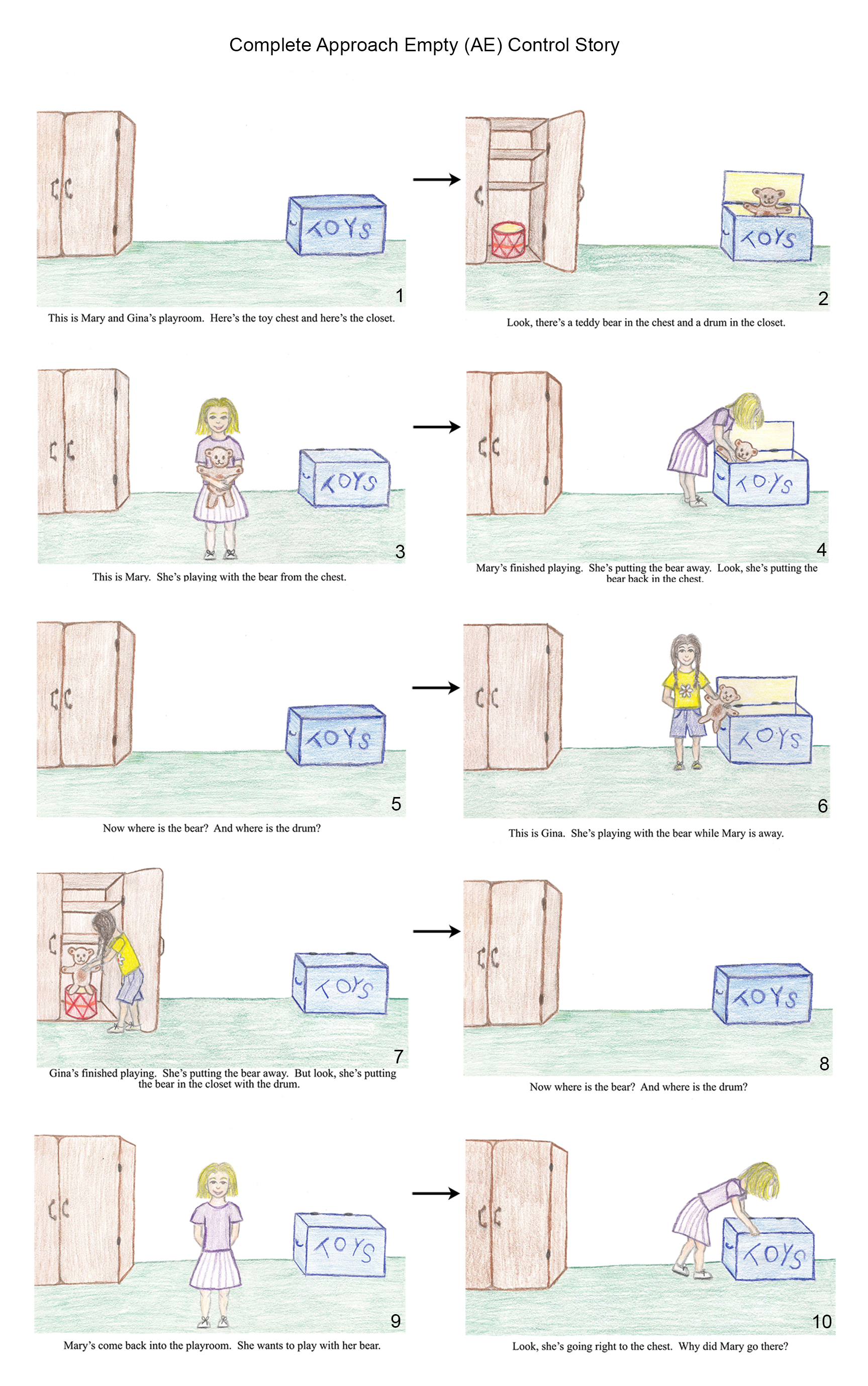

Supplement: Figure S2 — Complete Approach Empty Location (AE) control story. All ten panels and accompanying text are pictured. Note that this the text used for the desire-statement-present version of the stories; the text for the desire-statement-absent version of the stories was identical, except that the phrase “She wants to play with her [target object]” on panel 9 was removed. See Text S1 for the complete text from both versions of this story. (TIF) [file pone.0072835.s002.tif]
