# Supplementary material for: Theory of Mind in the Wild: Toward Tackling the Challenges of Everyday Mental State Reasoning
Source: PLoS One. 2013 Sep 12;8(9):e72835. doi: 10.1371/journal.pone.0072835 (PMC3771964; doi:10.1371/journal.pone.0072835)
Supplement: Text S1 — Full text of the explanation task stories depicted in Figures S1 and S2. The numbers refer to the story panels on which the text appears. The italicized text on panel 9 was included in the desire-statement-present version of the stories and removed from the desire-statement-absent version. (DOC) [file pone.0072835.s003.doc]

Text of Figure S1. Complete Approach Distracter Object (ADO) Story.

1. This is Jill and Dave’s playroom. Here’s the closet and here’s the desk.
2. Look, there’s a toy fish and a kite in the closet, but nothing behind the desk.
3. This is Jill. She’s playing with the fish from the closet.
4. Jill’s finished playing. She’s putting the fish away. Look, she’s putting the fish back in the closet with the kite.
5. Now where is the fish? And where is the kite?
6. This is Dave. He’s playing with the fish while Jill is away.
7. Dave’s finished playing. He’s putting the fish away. But look, he’s putting the fish behind the desk.
8. Now where is the fish? And where is the kite?
9. Jill’s come back into the playroom. She wants to play with her fish.
10. Look, she’s going right to the closet. Why did Jill go there?

Text of Figure S2. Complete Approach Empty Location (AE) Story.

1. This is Mary and Gina’s playroom. Here’s the toy chest and here’s the closet.
2. Look, there’s a teddy bear in the chest and a drum in the closet.
3. This is Mary. She’s playing with the bear from the chest.
4. Mary’s finished playing. She’s putting the bear away. Look, she’s putting the bear back in the chest.
5. Now where is the bear? And where is the drum?
6. This is Gina. She’s playing with the bear while Mary is away.
7. Gina’s finished playing. She’s putting the bear away. But look, she’s putting the bear in the closet with the drum.
8. Now where is the bear? And where is the drum?
9. Mary’s come back into the playroom. She wants to play with her bear.
10. Look, she’s going right to the closet. Why did Mary go there?
